# Green Strategies for Molecularly Imprinted Polymer Development

**DOI:** 10.3390/polym10030306

**Published:** 2018-03-12

**Authors:** Raquel Viveiros, Sílvia Rebocho, Teresa Casimiro

**Affiliations:** LAQV-REQUIMTE, Departamento de Química, Faculdade de Ciências e Tecnologia, Universidade NOVA de Lisboa, 2829-516 Caparica, Portugal; rfv17327@campus.fct.unl.pt (R.V.); s.rebocho@campus.fct.unl.pt (S.R.)

**Keywords:** molecular imprinting, supercritical carbon dioxide, ionic liquids, deep eutectic solvents (DESs), ultrasound-assisted, microwave synthesis

## Abstract

Molecular imprinting is a powerful technology to create artificial receptors within polymeric matrices. Although it was reported for the first time by Polyakov, eighty-four years ago, it remains, nowadays, a very challenging research area. Molecularly imprinted polymers (MIPs) have been successfully used in several applications where selective binding is a requirement, such as immunoassays, affinity separation, sensors, and catalysis. Conventional methods used on MIP production still use large amounts of organic solvents which, allied with stricter legislation on the use and release of chemicals to the environment and the presence of impurities on final materials, will boost, in our opinion, the use of new cleaner synthetic strategies, in particular, with the application of the principles of green chemistry and engineering. Supercritical carbon dioxide, microwave, ionic liquids, and ultrasound technology are some of the green strategies which have already been applied in MIP production. These strategies can improve MIP properties, such as controlled morphology, homogeneity of the binding sites, and the absence of organic solvents. This review intends to give examples reported in literature on green approaches to MIP development, from nano- to micron-scale applications.

## 1. Introduction

Green chemistry or sustainable chemistry can be defined as the design of chemical products and processes through the utilization of a set of principles able to reduce or eliminate the use or generation of hazardous substances. There are 12 principles of green chemistry and 12 principles of green engineering that should be followed to meet sustainability. These principles can be applied over the whole life cycle of a product, from its design, manufacture, and use, to its final disposal [1,2].

Herein we give an overview of how these green principles are being applied to the field of molecular imprinting. Many advances in the synthetic approaches have been made in the last years, however, molecular imprinting methodology still uses high quantities of solvents and intensive process steps, and this has, in our opinion, contributed for a difficult process adaptation and application of molecularly imprinted polymers (MIPs) at industrial scale, and hampered their wide use as real alternatives as affinity materials. Examples reported in literature will be shown, where green technologies and alternative solvents are used in MIP synthesis, as well as, tools for MIP design that can significantly decrease time and laboratory costs in MIP optimization.

In molecular imprinting, the formation of interaction complexes between the molecule of interest (template) and the functional monomers in the presence of a crosslinker and a porogen solvent, enable the production of specific sites within the polymeric material that are physically and chemically complementary to the template. After removing the template, the cavity is able to selectively bind the molecule in the final MIP application. The recognition mechanism can be mediated by different interactions, such as weak non-covalent hydrogen bonding interactions, ion-pairing, hydrophobic, or dipolar interactions. In addition, MIPs can be prepared according to different approaches, i.e., non-covalent, covalent, and semi-covalent, which differ in the way the template interacts with the functional monomer and with the polymeric binding sites in the final application.

Conventional MIP production techniques include polymerization in bulk, precipitation, emulsion, multi-step swelling, suspension, gelation, etc. The final particle sizes can vary from nano- to micron-sized particles, from irregular to completely spherical particles. MIPs can also be obtained in different final structures depending on the final application: membranes, fibers, core-shell particles, etc.

The affinity properties of MIPs are well recognized. MIPs are robust and present binding affinity constants similar to natural molecules [3]. Nevertheless, they also have some disadvantages. When they are prepared in bulk, the polymer must be crushed and sieved in a very time-consuming process, where very irregular particles are obtained. Conventional techniques also use high quantities of organic solvents. Water could be a good alternative, however, it can typically form strong interactions with the template and/or the monomers, and this would destabilize the formation of the complex and interfere in the formation of the specific sites. The use of green alternative solvents can overcome many of the disadvantages of MIP traditional production methodologies.

## 2. Green Strategies in MIP Development

### 2.1. Supercritical Fluid Technology

Supercritical carbon dioxide (scCO_2_) is a green alternative to replace typical organic solvents used in polymerization reactions. Indeed, scCO_2_ has several advantages, such as being inexpensive, non-toxic, non-flammable, inert, odorless, can be easily removed without any additional energy input, and can be recycled [4]. Moreover, CO_2_ is available in high purity as a sub-product from industry, and also, the price of CO_2_ is expected to decrease in the forthcoming years [5]. Thus, CO_2_ is a sustainable solvent full of potential to be used either in research area or industry, with an easy scale up. Moreover, CO_2_ properties can be easily tuned by simply adjusting pressure and temperature. CO_2_ has an easily obtained critical point (*p*_C_ = 73.8 bar; *T*_C_ = 31.1 °C) above which there is no longer vapor-liquid equilibrium, becoming the so-called supercritical fluid. Here there is a significant change in density by tuning pressure and temperature, and therefore, in all the properties that are density dependent, such as solubility.

Above its critical point, CO_2_ combines the best properties of gas and a liquid, such as gas-like diffusivity and viscosity, and a liquid-like density, leading to a high mass transport capacity and a very high diffusion coefficient compared to the liquid [6]. scCO_2_ is apolar and has a low dielectric constant, making it a suitable solvent not only for nonpolar molecules with low molecular weight, but also for small polar molecules such as methanol and vinyl monomers. A co-solvent can be added to tune its polarity. In addition, it has near-zero surface tension, which makes scCO_2_ an excellent medium for cleaning/extraction without damaging the structures to be cleaned.

Polymers, in particular with high molecular weight, have low solubility at mild conditions (<100 °C, <1000 bar) with the exception of fluoropolymers and polysiloxanes that show good solubility in scCO_2_, due to weak dipole-induced dipole interactions and flexible backbones, respectively [7,8]. Interestingly, the introduction of carbonyl groups on the polymer chains can increase, remarkably, the solubility of the polymer by weak interactions.

Polymer synthesis in scCO_2_ brings many advantages compared with traditional organic solvents, such as that scCO_2_ is aprotic and has high mass transfer and diffusivity which is not achieved easily using organic solvents, polymers are obtained by simply depressurization, have low toxicity, and low cost of preparation, making scCO_2_ an interesting solvent to replace commonly used organic solvents [9].

Another feature of scCO_2_ is that it can induce plasticization or melting of polymers (homopolymers, copolymers, and polymer blends) under a much lower temperature than their glass transition (*T*_g_) and melting (*T*_m_) temperatures, which have a strong impact on their mechanical and thermophysical properties [10,11,12]. scCO_2_ has already proved to be a versatile solvent reaction medium to produce polymers [13,14,15,16,17,18,19], as impregnation medium of polymers [20,21,22,23], as anti-solvent to produce solvent-free 3D structures [19,20,21,22], and as spray drying agent [23,24], etc.

Most commercially available monomers are soluble in scCO_2_, namely those typically used in MIP synthesis. MIPs developed using this green solvent are typically obtained as dry free-flowing powders, pure, sterile, in high yield, with narrow particle size distribution, without the need of any further drying or purification steps, ready-to-use, and easy-to handle [18,19], in opposition to traditional bulk methods, where MIPs need to be ground and sieved before use, leading to irregular particles and destruction of affinity sites [20].

The molecular imprinting method relies on the crosslinking of the growing polymer “freezing” the structure around the template molecules. The synthesis of a highly crosslinked polymer in scCO_2_ was reported for the first time in 1999, by Cooper et al. [21] in which non-porous poly(divinylbenzene) microspheres were obtained by heterogeneous polymerization with a final average particle size diameter between 1 and 5 µm.

The first MIP produced under scCO_2_ was reported by Duarte et al. [22] in 2006 for drug delivery purposes. Molecularly imprinted poly(diethylene glycol dimethacrylate) (polyDEGDMA) was produced by free radical polymerization (FRP) in scCO_2_ using a carboxylic acid end-capped perfluoropolyether oil as stabilizer for two different template molecules, salicylic acid and acetylsalicylic acid, in different concentrations. An impregnation step was further performed, and the controlled released of the systems were evaluated. Discrete spherical polymer particles with 1.7 μm diameter were obtained, and a correlation between the quantity of template used in MIP synthesis and the percentage of impregnation was achieved. In the same year, Ye et al. [23] shared with the scientific community the preparation of MIP nanoparticles via non-covalent imprinting strategy, using methacrylic acid (MAA) as monomer, divinylbenzene (DVB) as crosslinker, and propranolol as template molecule by heterogeneous polymerization, obtaining discrete crosslinked polymer particles with an average particle size around 100 nm.

Table 1 summarizes MIPs developed in scCO_2_ for several applications. Boc-l-tryptophan-MIP was synthesized by free radical polymerization in scCO_2_ for chiral separation [24]. Poly(ethylene glycol dimethacrylate) and poly(*N*-isopropylacrylamide-*co*-ethylene glycol dimethacrylate) based MIPs were obtained as micron-sized particles, in high yields (∼99%, determined gravimetrically) and ready to be slurry packed into HPLC blank column. Materials were evaluated as stationary phases for the enantiomeric separation of l- and d-tryptophan, and the polymeric materials were shown to be suitable for chiral separation for example in moving bed chromatography.

A MIP with affinity for flufenamic acid (FA) was developed for controlled drug release, by FRP using MAA and *N*-isopropyl acrylamide (NIPAAm) as monomers, and ethylene glycol dimethacrylate (EGDMA) as crosslinker [25]. The effect of crosslinking degree and template/monomer ratio were evaluated. Poly(MAA-*co*-EGDMA) and P(NIPAAm-*co*-EGDMA) were obtained as micron-sized agglomerates of nano-primary smooth surfaced particles, in high yields and as dry powders. Further, scCO_2_-assisted impregnation of FA showed that the imprinted matrices were able to uptake higher amounts of FA, 101.5 mg drug·g^−1^ polymer, compared to control non-imprinted polymer (NIP), 50.5 mg drug·g^−1^ polymer. In another work, ibuprofen (IBU)-imprinted polymer was synthetized in which EGDMA was used as crosslinker and 2-(dimethylamino)ethyl methacrylate (DMAEMA) was used as functional monomer with pH-sensitive character [26]. A lower crosslinking degree polymeric matrix (20.2 wt %) compared to the typical 80–90%, was used in a mimicked real scenario at two different pHs. MIPs were obtained as agglomerates of well-defined particles with particle size approximately of 1 μm, in high yields (∼85%, determined gravimetrically) as dry and free-flowing powders. MIP showed a higher molecular recognition towards the template, 33.1 wt % of IBU, compared to 10.2 wt % for the NIP polymer. 

In the last years, bisphenol A (BPA) has been the focus of a widespread interest because of its presence and accumulation in the environment, especially in the water, but is found also in scaring levels in the amniotic liquid of pregnant women. It is an endocrine disruptor that is commonly used in the production of polycarbonate plastics and epoxy resins [27], and it is known that the exposition to BPA increases the risk of cancer [28] and chronic diseases, such as diabetes [29]. BPA-imprinted polymer synthesis in scCO_2_ is reported in the literature by Soares da Silva et al. in 2012 [18,19]. MIPs were produced by FRP, using two different strategies: non-covalent and semi-covalent imprinting. In the first approach, MAA was used as functional monomer and EGDMA as crosslinker, while in the second approach, bisphenol dimethacrylate (BPADM) was used, a monomer containing BPA in its structure, and EGDMA as crosslinker. In the semi-covalent imprinting strategy, BPA was then cleaved from the polymer by hydrolysis with tetrabutylammonium hydroxide (*n*-Bu_4_NOH), also in scCO_2_. Both polymers were obtained as aggregates of discrete nanoparticles of smooth surface and as dry powders in high yields (>90%, determined gravimetrically). Non-covalent imprinted polymer could adsorb 450 µmol·g^−1^ from a BPA aqueous solution containing 300 µmol·L^−1^, while semi-covalent imprinted polymer could adsorb 98 µmol·g^−1^ from a BPA aqueous solution containing 50 µM when static binding tests were performed in both polymers.

Aspirin (AS) and acetaminophen (AAP)-imprinted polymers were prepared by Byun et al. [30] in 2013. There is an increasing concern with aquatic environment pollution [31] by active pharmaceuticals which can cause potentially liver damage [32] and cancer [33]. In this work, MAA or 4-vinylpyridine (4-VPy) were used as monomers, methyl methacrylate (MMA) as third monomer, and EGDMA as a crosslinker by three different strategies: bulk, emulsion, and scCO_2_-assisted polymerization. Fine MIP particles with average size of 250–300 nm were obtained. 4-VPy-based polymer was shown to be more efficient in binding capacity, adsorbing larger amounts of templates and showing higher imprinting factor (IF) value. In this case, scCO_2_-assisted polymerization was shown to be more efficient at selectively separating and detecting templates than the other polymerization processes. BPA and 2,4-dichlorophenoxyacetic acid-imprinted polymers were also reported by Byun et al. [34], in which MIPs were prepared by dispersion polymerization in scCO_2_, using MAA as functional monomer, EGDMA as the crosslinker, and poly(heptadecafluorodecyl methacrylate) (PHDFDMA) as a dispersing agent, obtaining particles with 300 nm. BPA and 2,4-dichlorophenoxyacetic acid, an herbicide compound, are typically present in aqueous solutions. Both polymers confirmed their efficient binding properties.

Carbamazepine (CMZ)-imprinted polymer was also synthesized by Byun et al. [35] by dispersion polymerization in scCO_2_, using MAA as functional monomer, EGDMA as the crosslinker, and PHDFDMA as a dispersion agent for selective separation of CMZ from aqueous solutions. CMZ is a drug broadly used to treat epilepsy and bipolar disorders which has also been found in effluent waters because of inefficient removal from wastewaters [36,37]. For this reason, CMZ is becoming a hazard to health and environment, due to the difficulty to completely remove this compound from water. It easily reaches the surface water, and is discharged to other media when water is reused. These authors produced nano-sized fine imprinted particles and evaluated the effect of pH and temperature. pH 7 and 20 °C were the conditions where the CMZ adsorption was the highest. They showed that CMZ-imprinted polymer is suitable for the selective separation of CMZ from aqueous solutions.

In 2014, labdanolic acid (LA)-imprinted polymer was reported by Martins et al. [38] for use on the LA purification from the acidic crude oil of *C. ladaniferus*. LA is a natural product from a Portuguese natural resource, and can be potentially used in medicine due to its relevant biological properties, such as anti-mutagenic and anti-inflammatory properties [39]. Several imprinted polymers were produced in scCO_2_ by FRP, such as poly(DMAEMA-*co*-EGDMA), poly(MMA-*co*-EGDMA), poly(2Vpy-*co*-EGDMA), and poly(HEMA-*co*-EGDMA), towards LA. Poly(DMAEMA-*co*-EGDMA) copolymer exhibited the best performance on the LA purification; when the acidic crude extract containing 15% of LA was passed two times through the column, 34% of LA could be selectively recovered. The results suggested that the choice of the monomer was crucial to the recognition performance of the system. In the same year, Ferreira et al. [40] reported the production of dibenzothiophene sulfone (DBTSO_2_)-imprinted polymer in scCO_2_ by FRP, for selective removal of the oxidized products from diesel. MAA-EGDMA copolymer was produced and obtained as an easy-to-handle white dry powder which was easily packed in the glass column to evaluate its performance. A model diesel solution containing DBTSO_2_ (23.1 mM) was passed through the MIP-glass column loaded with 200 mg of polymer, and 69.9% of DBTSO_2_ was retained. Additionally, the reusability of the columns was also evaluated for three consecutive cycles, and the retained ability increased with the reuse cycle, from 41.8 to 47.5%.

Gallic acid (GA)-MIP was reported this year by Byun et al. [41] for selective removal of GA from a natural matrix that has been extensively evaluated for antitumor activity [42]. In this manner, GA-imprinted polymer was synthesized by FRP in scCO_2_ using MMA as a third monomer, MAA as a functional monomer, EGDMA as a crosslinker, and THF as a porogen. The effect of crosslinking degree was also studied, and the average particle size of MIP particles prepared ranged 150–200 nm. It was shown that the MIPs’ adsorption capacity increased with the addition of up to 6 mol of EGDMA, however, for higher amounts the opposite trend was observed.

Recently, Viveiros et al. [43] described acetamide (ACET)-imprinted polymers for API purification processes. ACET is a pharmaceutical impurity present at low traces in API post-stream reactions [44] and has potential genotoxic behavior, which means that it can induce human diseases [45]. Copolymers were produced by FRP in scCO_2_, using two different monomers, MAA and MAM, and the effect of addition of a co-solvent, ACN, in the polymer synthesis was evaluated. Static and dynamic binding and selectivity tests were performed with ACET, benzamide (BENZ), and pivalamide (PIV) solutions. MAM-based MIP revealed the best performance, adsorbing 2.21 mmol·g^−1^ of ACET, when ACN was used as co-solvent in the polymerization step. MIPs also presented a higher selectivity for ACET than for BENZ and PIV.

2.1.1. 3D-Imprinted Porous Structures in scCO_2_

3D-imprinted materials have been a steadily growing field allowing to build other polymer geometries. In 2007, Zhang group [48] reported the use of poly(styrene-*co*-maleic acid) (PSMA) for molecularly imprinted membranes prepared by phase inversion in scCO_2_ for uracil (URA). URA-imprinted membrane was prepared using 15 wt % of PSMA and 2 wt % of URA in DMF, DMSO, and NMP solvents. Membranes were prepared at two different temperatures, 35 and 50 °C, and the system was slowly depressurized. URA was extracted from membrane with 1 vol % acetic acid solution, and after that, a large quantity of water for 1 week at 30 °C was passed through, and in the end, vacuum dried. The solvent used was shown to have a strong impact on membrane development, which could be confirmed by SEM. Membranes prepared at 50 °C presented higher affinity than membranes prepared at 35 °C, which corresponded to 12.6 and 9.2 μmol·g^−1^, respectively. Selectivity studies were performed using binary substrate solutions containing each of URA/DMURA, URA/thymine, and URA/cytosine at 2 μM. The results suggested that the URA imprinted membrane was effectively prepared.

Four years later, Zhang et al. [49] described oleanolic acid (OA)-imprinted membranes for OA extraction purposes. Oleanolic acid is a typical ingredient of medicinal plants, having anti-inflammatory properties [50]. PA6/PSMA-OA molecularly imprinted composite membranes were produced by phase inversion method in supercritical CO_2_ using polyamide-6 (PA6) as base membrane and poly(styrene-*co*-maleic acid) (PSMA) obtained by the conventional method. The mass ratio between PSMA and OA ranged from 3:1 to 8:1, and temperature and pressure from 35 to 50 °C and 12 to 17 MPa, respectively. As a result, the specific conditions mass ratio of PSMA and OA 6:1, 40 °C, and 15 MPa, had the best performance, where the imprinted membrane could adsorb 50.4% and 96.1% of OA, respectively. Therefore, OA-imprinted membranes produced using scCO_2_ provided a feasible method to selectively separate OA from other compounds.

Molecularly imprinted composite membranes were reported by Soares da Silva et al. [18,19] for poly(MAA-*co*-EGDMA) and poly(BPADM-*co*-EGDMA)-MIPs for BPA that had been obtained by non-covalent and semi-covalent imprinting, used in membrane preparation by scCO_2_ assisted-phase inversion. The strategy was to confer affinity to the membrane by incorporating the MIP particles within the membrane. For membrane preparation, 30 wt % of polymer blend consisting of 70:30 PMMA/poly(MAA-*co*-EGDMA) and PMMA/poly(BPADM-*co*-EGDMA) were dissolved in DMF. The inversion process occurred for 3 h at 45 °C, and at the end, the system was slowly depressurized over 20 min. The non-covalent imprinted and semi-covalent imprinted membranes were obtained as thin and homogeneous layers, and could adsorb 1.3 and 2.1 times more BPA, respectively, than the corresponding control membranes.

#### 2.1.2. MIP-Supported Materials Preparation in scCO_2_

MIP-supported materials have been shown to be a very appealing area, were the properties of the core materials can be improved or changed, which can have a strong impact on the materials’ performance, such as in the production of a MIP layer on supports as silica, titanium oxide, quantum dots or polymers, among others.

Supercritical CO_2_-assisted synthesis of an ultrasensitive amphibious quantum dot-molecularly imprinted sensor to BPA was reported by Lourenço et al. [51] where CdTe quantum dots were previously synthesized and functionalized in a conventional way with mixed thiols, to confer both stability and reactivity. Then, the MIP containing the quantum dot was produced in scCO_2_, using MAA as functional monomer, EGDMA as crosslinker and BPA as template, by FRP. Quantum Dots (QDs) as spherical discrete particles were obtained with particle size diameter of 5–10 nm with a low degree of aggregation. On the other hand, CdTe@MIPs presented larger sizes, due to their surface polymer coating. QDs could be successfully incorporated within molecularly imprinted matrices using supercritical carbon dioxide, and sensing properties of CdTe@MIP towards BPA was evaluated by fluorescence spectroscopy by using only 2 mg of material in the presence of different concentrations of BPA aqueous solutions, up to 500 nM. CdTe@MIP demonstrated a well-defined highly sensitive quenching of the fluorescence, showing it to be a promising candidate for the development of BPA sensors (4 nM detection limit).

**Figure 1:**
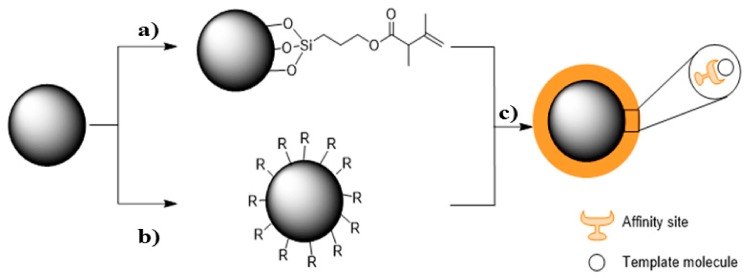
Scheme for the production of MIP-layered silica beads: (**a**) 3-(Trimethoxysilyl) propyl methacrylate (MPS)-functionalization in scCO_2_; (**b**) plasma functionalization of silica beads, in which *R* are the radicals produced; (**c**) MIP-layered core-shell beads produced in scCO_2_. Reproduced from reference [52] with permission from Elsevier.

Casimiro’s group has developed large MIP-layered core-shell beads using supercritical carbon dioxide technology, for gravity-driven purification systems to remove impurities from pharmaceutical crude mixtures (Figure 1) [52]. scCO_2_ was used to pre-functionalize core-shell particles and perform the MIP-layer synthesis over large silica beads (75–200 μm), where MAA was used as monomer, EGDMA as crosslinker, and ACET as a model pharmaceutical impurity. Particles were loaded on a SPE column, and their performance evaluated in gravitational mode using a 250 ppm ACET solution in ACN. Imprinted particles could remove 550 μg _ACET_·g_support_^−1^, 24% more than the control particles.

### 2.2. Ionic Liquids (ILs) and Deep Eutectic Solvents (DES)

Ionic liquids (ILs) are organic salts, composed by an anion and a cation, that remain liquid near room temperature. The term “designer solvents” is frequently applied to these compounds, due to their particularly versatility: through the combination of different cations and ions, ILs can be obtained with very different physical and chemical properties, such as melting point, viscosity, density, solvation ability, and hydrophobicity [53]. ILs also have typically excellent thermal stability and are non-flammable [54]. One of the most important characteristics of ILs is their negligible vapor pressure, which means that they are essentially non-volatile, and can be contained, completely recycled, and used several times, which is a great advantage over volatile organic compounds [55].

However, their high cost is still an issue, and also, the toxicity effect on humans/environment of most ILs is still unclear [56,57], which leads to the question, how green are these solvents? Nevertheless, this drawback can be overcome by choosing an infinite variety of cations and anions, enabling the specific design of less toxic ILs.

**Figure 2:**
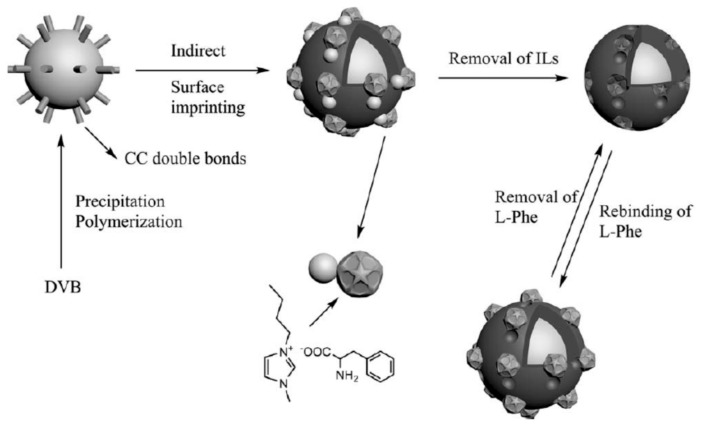
Protocol for the synthesis of MIPs for l-phenylalanine using 1-butyl-3-methylimidazolium α-aminohydrocinnamic acid salt [BMIM][Phe] as template. Reproduced from reference [67] with permission from John Wiley and Sons.

Since the nineties, the number of publications in this area increased exponentially [58] with applications in many different fields, including the production of MIPs, wherein ILs started to be used as solvents [59,60,61,62,63,64,65], and further with other functionalities, such as templates (Figure 2) [66,67,68,69], monomers [70,71,72], crosslinkers [73] or additives to polymer reactions.

It has already been shown that the use of ILs in MIP synthesis can accelerate the process, also improving the selectivity and adsorption of the template [74,75].

#### 2.2.1. ILs as Solvents (Porogenic Solvents)

MIP synthesis towards *trans*-aconitic acid, using ILs as porogenic solvents, was reported by the first time by Booker and his co-workers, in which [Bmim][BF4] and [Bmim][PF6] were used, under photochemical and thermal conditions. MAA was used as monomer and EGDMA or TRIM as crosslinker. The impact of ILs on bulk polymerization was evaluated, and both ILs revealed a faster synthesis of polymer microspheres (*d*_p_ < 200 nm). In the performance of the materials, MIP produced using [Bmim][BF4] and thermal initiator was shown to be quite selective [59]. Because of the tunable nature of the ILs, there is the possibility to overcome the lack of template solubility at the beginning of the polymerization, such as, for example, opiates that show very low solubility in conventional organic solvents. Also, ILs properties can be fine-tuned for each specific application, that is why they are called designer solvents.

Later on, the same group [63] performed the same study, testing not only [Bmim][BF4] and [Bmim][PF6], but also two other different ILs, [Hmim][PF6], [Omim][PF6], varying the length of the alkyl chain, on MIP preparation for propranolol. They could see that the length of alkyl chain length had strong impact on solvent viscosity, meaning that [Hmim][PF6] and [Omim][PF6] showed the low binding performance, because they increase the IL’s viscosity.

#### 2.2.2. ILs as Monomers

ILs can be used as functional monomers in MIP production. Interesting applications for the incorporation of IL monomers into polymers have been published [70,71,72], but the range of possible structures with ILs is so broad that it still has a long way to be explored.

To illustrate the potentiality of using ILs as monomers, Luo et al. [71] described the use of 1-(α-methyl acrylate)-3-methylimidazolium bromide (1-MA-3MI-Br) IL as a functional monomer, for MIP production for caffeine. The performance of this system was compared with MAA-MIPs, in which the first one showed a superior adsorption capacity. Furthermore, the adsorption capacity of caffeine using the IL-MIPs in water showed the highest value, when compared with methanol, ACN, and methylene dichloride. So, this is a very promising result, which demonstrates IL’s potential as monomer, and in addition, 1-MA-3MI-Br-MIPs demonstrated a good compatibility with aqueous environments.

Another example found in the literature is the preparation of IL-MIPs by surface imprinting. Ding et al. reported the production of acrylamide modified multi-walled carbon nanotubes (MWCNTs-AAm)-MIPs (MWCNTs@BSA-MIPILs) [72] with affinity to BSA, using four different allyl-functionalized ILs as monomers. The selective recognition ability provided by the IL-based MIPs was higher for BSA over analogue molecules such as HSA, Lys, Try, and BHb. Once again, the results showed that ILs were water-compatible, which is an important parameter in biomacromolecules’ stabilization.

#### 2.2.3. ILs as Crosslinkers

The crosslinker plays a key role on the MIP structure by “freezing” the growing polymer around the template molecules, enabling the formation of the specific cavities. The benefit of using ILs as crosslinkers has recently been highlighted with the development of MIP polymeric nanoGUMBOS (nanoparticles derived from a group of uniform materials based on organic salts) using four ILs as crosslinkers. The vinylimidazole-based IL crosslinkers were synthesized and subsequently explored to develop MIPs for l-tryptophan. Each of the crosslinkers was used in the synthesis of the MIPs, and their performance was evaluated using chiral recognition. High uptake values for l-tryptophan were obtained compared to d-tryptophan. These materials were shown to be suitable for chiral recognition, and the results envisaged the utility of these materials for imprinting aqueous templates, such as biological targets for theranostic agents [73].

#### 2.2.4. ILs as Templates

Li and co-workers reported an interesting approach in the development of “dummy” l-phenylalanine MIP microspheres by using [Bvim][Phe] IL with both template and functional monomer functionalities [66]. The 4-VPy and EGDMA were used as co-monomer and crosslinker, respectively, in the preparation of surface-imprinted polymer on poly(DVB) microspheres. The template and the cation vinylimidazolium formed ion pairs, in which the IL is well located inside the imprinted cavities. The results showed that polymerizable ionic liquid which was worked as dummy template could enhance the affinity and selectivity of MIP, promoting the development of MIPs for biomolecules.

#### 2.2.5. Deep Eutectic Solvents (DESs)

DESs are commonly designated as fourth generation ILs, which are basically mixtures of salts, that are obtained by the complexation between a hydrogen acceptor, such as nontoxic quaternary ammonium salt, choline chloride, and naturally-derived uncharged hydrogen bond donors, as alcohols, carboxylic acids, amides, amines, urea, and glycerol. DES can also be made by compounds that are non-ionic, which is obviously an advantage over the conventional ILs. The main advantages are low cost, biodegradability, no need of additional purification, and the low toxicity of anions and cations used [76,77].

Li et al. [78] reported the use of a DES as an auxiliary solvent to enhance the affinity and selectivity of MIPs towards chlorogenic acid (CA). DES was synthetized using choline chloride (organic cation) and glycerol (inorganic) (1:2 molar ratio). DES-NIPs and NIP and MIP without DES were prepared for comparison at identical conditions. The DES-MIPs exhibited the most promising SPE recoveries of CA, 72.56% compared with DES-NIPs (64.79%), MIPs (69.34%), and NIPs (60.08%), respectively, for the purification of chlorogenic acid from honeysuckle.

In addition, Liu and co-workers [79] explored the application of magnetic DES as a new type of functional monomer in molecular imprinted technology. DES was synthetized using choline chloride and methacrylic acid (1:2 molar ratio), and the magnetic ability was provided by Fe_3_O_4_. These affinity materials were designed to separate proteins using BHb as template molecule. The magnetic DES-MIPs could adsorb 184.10 mg·g^−1^ of BHb compared with analogue molecules such as Lys (101.97 mg·g^−1^), BSA (87.29 mg·g^−1^), and ovalbumin (18.44 mg·g^−1^), respectively.

### 2.3. Ultrasound-Assisted MIP Synthesis

Ultrasound-assisted polymer synthesis has been extensively used to enhance the reaction rate [80], taking advantage of the formation and collapse of small bubbles by the cavitation caused by ultrasonic energy, which increases the solubility of template and monomers, diffusivity, penetration, and transportation of species in the medium [81,82].

This technology brings several advantages in MIP synthesis when compared with traditional polymerization processes, because it promotes higher reaction rates, more homogeneous polymer chain growth, higher yields, and milder conditions (e.g., low reaction temperature) [83,84]. Furthermore, it can alter the binding site population distribution, and thus, the morphology of the final polymer [82]. 

Only a few works have reported on MIP synthesis by ultrasound-assisted polymerization. However, MIPs prepared ultrasonically presented binding properties similar or superior to the conventional methods [82,84,85]. The first MIP produced using ultrasound-assisted polymerization was reported in 2006 by Svenson [82], in which theophylline (THO)-imprinted polymers were produced using MAA, EGDMA, and AIBN as functional monomer, crosslinker, and free radical initiator, respectively, for chromatographic processes. THO is a drug extensively used in the treatment of asthma, even though its use has continually declined over the years, due to its side effects and toxicity [86]. Polymerization reactions were performed at 65 °C for 4 h using an ultrasonic bath operating at 35 kHz. Polymers were obtained similarly to conventional MIP protocols. A white monolith was obtained that was further crushed, ground, and sieved through a 63 µm sieve. Polymer was obtained as irregular particles with 60–65% yield. Moreover, the cavitation showed had no influence on polymers produced, however, less time was required for polymerization process and the binding characteristics of polymers obtained could be compared with MIPs prepared using more traditional protocols.

The encapsulation of magnetic nanoparticles by 17β-estradiol-imprinted polymers using ultrasonication-assisted synthesis was reported by Xia et al. [87] for fast removal of 17β-estradiol from aqueous environments. In this case, MAA was used as monomer, EGDMA as crosslinker, and AIBN as initiator, at 42 kHz, 65 °C for 2 h. The average particle size diameters of magnetic NIPs and magnetic MIPs were 200 and 300 nm, respectively. The use of ultrasound not only enhanced the polymerization rate and morphology of the nanoparticles, but also led to an increase in the number of free radicals, and thus, facilitated MIP growth around the magnetic nanoparticles. The adsorption capacity towards to 17β-estradiol was comparable to traditional approaches.

Ultrasound-assisted precipitation polymerization was also used in the production of caffeine–MIP supported on magnetic particles by Phutthawong et al. [88]. In this example, the affinity layer was produced using MAA as monomer, EGDMA as crosslinker, and BPO as initiator. Polymerization reactions were performed using an ultrasonic bath operating at 37 kHz, and kept at 60 °C between 0.5 to 4 h. Copolymers were obtained as agglomerates of particles with similar size. The yields of magnetic MIPs increased from 0.5 to 4 h (47.1–62.4%). Moreover, binding properties were evaluated in static mode for 16 h where magnetic MIPs produced ultrasonically for 4 h obtained the best result, adsorbing 11.14 times more than the magnetic NIPs. Affinity properties were also evaluated using THO as analogue molecule, and particles were shown to be very selective. The reusability of structures was finally assessed, and the adsorption amounts of caffeine were kept almost constant for five cycles. The same group [89] also described the caffeine-imprinted polymers using MAA as monomer, EGDMA as crosslinker, and BPO as initiator, by ultrasound-assisted precipitation polymerization, in which polymer microspheres in high yields and with narrow size distributions were obtained, with number average particle size diameter (*d*_n_) between 0.28 to 0.56 μm. Imprinted polymers prepared at 40 °C had the best caffeine binding performance, adsorbing 21.63 μmol·g^−1^ and with IF of 6.54.

MIP-coated Mn-doped ZnS quantum dots for specific fluorescent recognition of cocaine (COC) and metabolites for tracing drugs, were described by Chantada-Vázquez et al. [90]. Ultrasound technology allowed the production of MIP layer on QDs by precipitation polymerization, in which EGDMA was used as a functional monomer, DVB as crosslinker, and AIBN as initiator, to proceed the syntheses at 37 kHz for 4 h. Structures produced showed to be able to detect COC and the metabolites, benzoylecgonine (BZE), and ecgonine methyl ester (EME). Another group [91] reported MIP-layered magnetic supports towards to the same template-COC and metabolites, as BZE, cocaethylene (CE), and EME which were produced by ultrasonically-assisted polymerization. MIP syntheses were performed using EDGMA as functional monomer, DVB as crosslinker and AIBN as initiator under sonication conditions (37 kHz, 325 W, 30 °C) for 4 h. Then, MIP-magnetic particles could be obtained in an efficient way as agglomerates of spherical particles with approximately 50 nm of particle size diameter. Further, particles were packed on a solid phase extraction (SPE) column, showing selective behavior for COC and metabolites in urine samples.

These examples describe well the high potential of ultrasound-assisted technology in MIP synthesis. This technology has been also used as an additional step to traditional processes to assist the template desorption from the final MIPs at the end of the polymerization [92].

### 2.4. Microwave-Assisted MIP Synthesis

Microwave has emerged as an alternative in MIP synthesis, dramatically decreasing the time required in the polymerization process, typically less than 1/10 of the required conventional heating. [93]. Although the mechanism is still not well understood and remains a point of debate [94], the benefits of this clean technology are very clear, promoting homogeneous and rapid heat transfer through the reaction mixture, leading to higher reaction rates, short-term polymerization processes, and high yields [95].

Microwave-assisted MIP preparation is a fast and simple methodology to obtain crosslinked polymers with controlled morphology and good recognition properties [96]. MIP-based devices have recently been explored using this promising technology, which provides a clean and less time-consuming approach to produce powders, layers, membranes, or hollow fibers.

In 2009, Zhang et al. [93] reported the production of atrazine (ATR)-imprinted polymer layered magnetic particles by suspension polymerization using microwave heating, for tracing triazines on complex samples. MIP layered-magnetic particles were produced using MAA as monomer, TRIM and DVB as crosslinkers, AIBN as initiator, and water as a solvent media, using microwave heating for 120 min at 70 °C by increasing the temperature from room temperature in 3 min. Magnetic MIPs were obtained with uniform morphology and a narrow size distribution with an average particle size diameter ranging 100–200 µm. The results obtained were comparable to the magnetic MIP prepared by conventional heating. MIP particles showed to have superior binding ability and selectivity for triazines, exhibiting higher imprinted factors compared with traditional ones. The process had a dramatic reduction in the time of the polymerization reactions. When a dual-phase solvent system of *n*-hexane and water was used in the same polymerization system, the imprinting efficiency of ATR increased from 0.5 to 4.4, and the sensitivity for seven triazines (presented in tomato, strawberry juice, and milk) increased 3.1–6.6 times compared with pure aqueous medium. The results opened up the possibility for further use of MIP in other aqueous media, such as biological fluids [97].

Microwave induced caffeine-imprinted polymer polymerization was described by Turner et al. [98]. MAA, EGDMA, and AIBN were used as monomer, crosslinker, and initiator, respectively. Polymerization reactions were carried out in a microwave reactor, and heated at 150 W for 14 min at 60 °C. Agglomerates of nanoparticles were obtained, which were further packed on SPE columns, and the binding affinity and selectivity were evaluated with caffeine and THO. This microwave-assisted process reduced the preparation time by 70-fold when compared with conventional methods.

In another example, also reported by Zhang et al. [96], indole-3-acetic acid (IAA)-MIP layered magnetic beads were developed through suspension polymerization under microwave heating, for application in the isolation and enrichment of auxins from plant tissues. 4-Vpy and silylated β-cyclodextrin (β-CD) were used as monomers alone or as co-monomers, TRIM as crosslinker, and AIBN as initiator. IAA-MIP magnetic beads were obtained as homogeneous spherical shape particles with a medium diameter between 50 to 200 µm. The MIP layer was porous and presented a rough surface with high surface area. MIP–magnetic particles revealed higher binding capacity for IAA when both monomers (4-VP and silylated β-CD) were used. Kaempferol-MIP microspheres were also prepared by microwave-assisted polymerization, for the detection of kaempferol content in traditional Chinese medicines [99].

Other MIPs have been successfully produced using this green chemistry tool, with affinity to ractopamine [100], 2-amino-4-nitrophenol (4-NAP) [101], gibberellin acid 3 (GA3) [102], tetracycline [103,104], BPA [105], atrazine [106], enrofloxacin hydrochloride (ENRH) [107], tramadol [108], theophylline (THO) [109], resveratrol (RES) [110], baicalein (BAI) [111] and florfenicol (FF) [112], among others.

### 2.5. Solvent-Free and Solvent-Less MIP Preparation

Solvent-free strategies have been used in polymer synthesis, not only for environmental issues, but also to drastically reduce or eliminate the intensive use of organic solvents.

Zhong group [113] reported the solvent-free polymerization method to prepare levofloxacin (LOFX)-MIPs. LOFX is an antibacterial drug used in the treatment of respiratory diseases, such as bronchitis and pneumonia [114]. The frontal polymerization (FP) was carried out using MAA as monomer, DVB as crosslinker, sodium dodecyl sulfate (used to increase the viscosity of the reaction mixture), AAm as co-monomer, NaHCO_3_, and AIBN, in a thick-walled glass tube at 60 °C for 30 min. They evaluated the influence of LOFX and AAm concentrations in terms of IF and performance of the materials. The nano-sized LOFX-MIPs were obtained as dry polymers that were ground and sieved for further use. Then, batch binding and selectivity experiments were performed in methanol, where a higher adsorption capacity was achieved for LOFX, adsorbing 57 mmol·L^−1^. LOFX-MIP showed higher IF than conventional bulk and precipitation polymerization, 5.78 against 2.34 and 1.76, respectively. Thus, frontal polymerization provided a rapid, solvent-less and economical way to prepare MIPs, with significant lower energy consumption, also promoting high reaction rates in short time periods, from 10 to 30 min.

### 2.6. Electropolymerization-Assisted MIPs

Electropolymerization brings many beneficial aspects, since it allows the control of the thickness of the polymer layer. The polymer can be attached to the sensor surface with different types of geometries, and is compatible with combinatorial and high-throughput strategies [115,116]. In general, this approach is performed when MIP sensitive devices are developed, such as capacitive, electrochemical, voltametric, or amperimetric sensors.

The Malitesta group [116] described for the first time, the electrosynthesis of a glucose-MIP on the electrode of a quartz crystal. The electropolymerization of *o*-phenylenediamine was performed in acetate buffer (pH 5.2) and glucose by cyclic voltammetry in the range 0.0–0.8 V. The results showed that this is a feasible way to prepare biomimetic sensors for glucose. Also Panasyuk et al. [117] reported a phenylalanine-MIP layer on gold electrodes produced by electropolymerization of phenol as monomer. Polyphenol MIP monolayer with affinity towards phenylalanine was produced at room temperature by cyclic voltammetry at 0.3 V. The results showed a decrease on the electrical capacitance when increasing the template concentration. Cheng et al. [118] reported a glucose-MIP layer on gold electrode synthesized via electropolymerization of *o*-phenylenediamine in the presence of the template glucose, dissolved in acetate buffer solution (pH 5.18) by cyclic voltammetry, and the resulting sensor was shown to be stable and reproducible, exhibiting higher selectivity to glucose than for ascorbic acid or fructose.

Electropolymerization is being widely used in the synthesis of MIPs, especially advantageous in the preparation of sensing devices. Many other templates have been used, such as cholesterol [119,120], fluorescein, rhodamine, and 2,4-dichlorophenoxyacetic acid (2,4-D) [121], fenvalerate [122], caffeine [123], morphine [124], paracetamol [125], 4,6-dinitro-*o*-cresol [126], ascorbic acid [127], sulfamethoxazole [128], triclosan [129], folic acid [130], BPA [131], naproxen, paracetamol, and THO [132], methyl-parathion pesticide [133], bovine hemoglobin (BHb) [134], and estradiol (E_2_) [135]. 

Cai et al. [136] reported human ferritin and human papillomavirus-derived E7 proteins MIP-layered carbon nanotube tips via an electropolymerization method for protein detection. The polyphenol (PPn) was deposited at carbon nanotube tip surface by cyclic voltammetry in PBS containing phenol between 0.0 and 0.9 V, and further, the protein was added to the solution, and more five cycles of voltage were applied. As a result, MIP sensor was obtained as nano-sized particles at the sensor surface, providing a label-free electrochemical detection with ability to selectively recognize the proteins over calmodulin.

### 2.7. Computational Tools on Rational Design of MIPs

Computational tools have been increasingly used to replace combinatorial screening or trial-and-error methods to produce MIPs. Combinatorial synthesis and screening were the most used strategies for rational design of molecularly imprinted polymers [137]. However, combinatorial synthesis has some drawbacks. For example, to study two different parameters using 100 monomers, it would be necessary to prepare more than one thousand polymers. Molecular modeling and thermodynamic calculations are a potential solution for this problem [138]. Molecular mechanics (MM) provides a qualitative description of the system based on potential energy, and allows the calculations to be run in an easy way. Atoms are considered as balls which have a specific radius and a string. The atom sizes, bond geometry, and strength are determined using empirical data from X-ray crystallography and NMR experiments [139]. AMBER, HyperChem 501, GROMACS, Gaussian [139], MOE, RasMol, QMol, Raster 3D, and AGM Build are some of most used MM software packages used in several applications. The main limitation of computational design of MIPs is based on thermodynamic calculations [140] for multicomponent systems. However molecular modelling of complex systems, including interactions of polymers with template, solvent, and other molecules are difficult, due to the requirements of computational workstation [139]. Some drawbacks are associated with these programs, since some of them cannot generate a clear result. In the end, the results have to be crossed, requiring a lot of time to organize data and make conclusions.

The Piletsky group developed a methodology that can greatly simplify this process, because instead of modelling the polymer, they focus on the monomer mixture and the interactions between monomers, template, and solvent, using the program SYBYL^TM^, significantly reducing the computational load [6,9,11]. SYBYL^TM^ is a user-friendly program typically applied in drug design [141]. The Pilestsky group successfully adapted this program to measure affinities between molecules involved in the MIP synthesis, performing rational MIP design [139]. In 2001, Piletsky et al. [140,142] reported for the first time the computational design of a MIP specific to ephedrine using SYBYL^TM^, where a virtual library containing all functional monomers was previously created and further screened using the LEAPFROG^TM^ algorithm against the template. The monomers which gave the highest binding scores, ITA, MAA, HEMA, AA, and 2-Vpy were selected to be experimentally used in the co-polymerization with EGDMA (crosslinker), chloroform, and THF as porogenic solvents, and 1,1′-azobis(cyclohexanecarbonitrile) as initiator, overnight at 80 °C. Copolymers were obtained as bulk material which were ground using a SL2 suspension grinder and wet-sieved to collect a final fraction of the polymer with particle size between 38 and 106 µm. The five MIPs were packed into HPLC columns, and their performances were evaluated. ITA and MAA-based MIPs showed strong interactions with ephedrine. As a result, a clear correlation was attained between the modelling results and the performance of the materials, meaning that the computational studies provided a useful tool to predict the affinity and specificity of the MIP mimicking real experiments.

In 2002, Chianella et al. [143] explored the use of a SYBYL-computational approach for rational design of MIPs towards cyanobacterial toxin mycrocystin-LR. The molecular modelling software uses a virtual library containing all polymerizable monomers that are screened against the target molecule. The best suitable monomer was the one which generated higher monomer-template binding energies. 2-Methyl-1-propanesulfonic acid (AMPSA) was selected as the best monomer, followed by imidazole-4-acrylic acid ethyl ester (UAEE), and MAA. Polymers were synthesized by FRP in DMSO using mycrocystin-LR as template, AMPSA, UAEE, and MAA as monomers, EGDMA as crosslinker, and 1,1′-azobis(cyclohexanecarbonitrile) as initiator, at 80 °C for 24 h. The copolymers were obtained as bulk material, which was ground and sieved to have, in the end, two final fractions obtained by using two sieves with mesh size of 63 and 45 µm. The performance of the synthetic MIP towards mycrocystin-LR could be compared with monoclonal and polyclonal antibodies. The results indicated that MIP showed higher affinity and comparable selectivity to the polyclonal antibodies with a detection limit of 0.1 µg·L^−1^, with beneficial features such as chemical and thermal stability over natural molecules. Selectivity was also evaluated with other toxin analogues which demonstrated very low performance. Computationally designed MIP was an efficient strategy to anticipate the affinity and specificity of the polymer.

The synthesis of MIPs for picolinamide (PAM), nicotinamide (NAM), isonicotinamide (*i*-NAM), *p*-hydroxybenzoic acid (*p*-BA), *p*-hydroxylphenylacetic acid (*p*-HBA), and *p*-hydroxyphenylpropionic acid (*p*-HPPA), in which MIPs were pre-designed using a computational approach—GAUSSIAN 94 quantum software—was reported by Wu et al. [144]. The GAUSSIAN predictions were focused on the monomer–template interaction in the pre-polymerization step. MAA was selected as the best monomer to PAM, NAM, and *i*-NAM, while AAm was selected as the best monomer for *p*-BA, *p*-HBA, and *p*-HPPA. The FRP reactions were performed using EGDMA as crosslinker, and AIBN as initiator, in chloroform and ACN as solvents. Then, copolymers were evaluated experimentally as chromatographic stationary phases, where PAM and *p*-BA based-MIPs showed a positive correlation between the binding energy and the capacity factor. The results showed that the monomer–template complex with highest binding energy promoted the most stable complex in the pre-polymerization stage. Meng et al. [145] developed a MOPAC-computational approach to optimize monomer composition of *p*-nitrophenyl acetate (*p*-NA)-MIP catalyst, creating a virtual library containing the intermediates of a lipase-catalyzed transesterification process. The binding energies of the intermediates were minimized using the semi-empirical MOPAC method, in which the most stable complex provided the best complex to perform the synthesis, and also led to the optimal catalytic performance.

Still in 2004, Turner et al. [146] explored SYBYL as a computational approach to select the best monomer-template complex to perform mycotoxin ochratoxin A (OTA)-MIP synthesis. The pre-computed results suggested the use of MAA and AAm as monomers, EGDMA as crosslinker, and 1,1′-azobis(cyclohexanecarbonitrile) as initiator in DMF solvent was a suitable reaction mixture to prepare the polymer. Polymers were obtained as monoliths, which were ground and sieved to obtain a usable fraction with range size 38–63 µm. OTA-MIP were shown to be able to bind specifically to OTA, especially in aqueous environment compared to organic solvents. Moreover, the binding properties were shown to be strongly dependent on pH and ionic concentration. Also, accordingly with results, the binding mechanism was shown to be dependent on the conformation of the polymeric binding pockets, and the presence of weak electrostatic interactions, which promoted a specific recognition.

Many other MIP systems have been optimized by using computational tools. Dong et al. [147] provided a GAUSSIAN computational approach to design THO-MIP, where the interaction between monomer and template were computationally evaluated. Wu et al. [148] reported the use of GAUSSIAN as a computational tool to simulate the MIP synthesis towards NAM and *i*-NAM. Pavel et al. [149] reported an atomistic model to design monomers and polymers for molecular imprinting of THO and its derivatives using molecular dynamics (MD) simulations to predict the binding energies. This approach was applied in five template molecules, THO, and its derivatives (theobromine, theophylline-8-butanoic acid, caffeine, and theophylline-7-acetic acid) and in the end, minimized structures were attained and stable complexes were obtained using MM. These authors also reported an extension of the previous work, where in this case, the solvent, ethanol, was added to molecular simulations [150]. They showed that electrostatic interactions had a strong impact on the formation of the molecular imprinting structures. Moreover, the simulated results showed that monomers or polymers interacted with template preferentially by –COOH or CH_2_=CH–. Additionally, it was verified that a template without functional side groups are suitable for molecular imprinting technology. Furthermore, the presence of solvent favored the stability of THO-monomer/polymer complex over its derivatives.

SYBYL computationally-designed MIPs towards to herbicide simazine for environmentally control of algae was also reported in 2005 by Piletska et al. [151]. Dineiro et al. [152] reported a GAUSSIAN computational approach for the rational design of homovanillic acid (HVA)-MIPs. Density functional theory (DFT) methodology was used to design MIPs, allowing the choice the most suitable monomer and solvent to prepare MIPs. A comparison between stabilization energies of the pre-polymerization adducts between template and functional monomer is the basis of this methodology. The same group [153] also provided an alternative approach, DFT-computational prediction and experimental evaluation for HVA-MIP, in which trifluoromethacrylic acid (TFMAA)/toluene was the best combination of functional monomer/solvent that promoted the most stable pre-polymerization adducts. Monti et al. [154] proposed a combination of MD, MM, docking and site mapping computational methods to design highly selective THO-MIP in ACN. MAA and MMA were used as monomers to simulate the final polymer. This methodology was able to predict the affinity and selectivity of MAA-MIP towards THO and analogue molecules (caffeine, theobromine, xanthine, and 3-methylxanthine). Dong et al. [155] reported a DFT-molecular simulation and experimental validation of the influence of solvent on adsorption capacity of THO-MIPs. Computational simulations indicated chloroform as the most suitable solvent to prepare THO-MIP compared to THF and DMSO. Liu et al. [156] described MMFF94 force field as a computational tool to evaluate the properties of paracetamol-MIPs. The binding energies between template and monomers can be calculated through this model, as well as the most possible conformations of template-monomer when they are interacting.

Nantasenamat et al. [157] proposed a quantitative structure-imprinting factor computational approach with application on MIP design, which correlates the template-monomer complex with the IF. The QSPR evaluation correlates the monomer-template structure with the IF based on quantum chemical descriptors derived from single point calculation at B3LYP/6-31G(d) of geometrically optimized structure at the HF/3-21G(d) level of theory. The analysis of MIP particles from the literature was used to compute the imprinting factor using molecular descriptors derived from charge density-based electronic properties of molecules in which reliable results were predicted with high correlation coefficients. In addition, the mobile phase, which can be aqueous, pH, dielectric constant of binary solvents, and ionic strength of aqueous buffers was shown to have a strong impact on the performance of the predictive QSPR model. This feasible methodology could serve as a basis for the development of software packages which are able to predict MIP performance before testing.

Yao et al. [158] reported the use of computational design by screening functional monomers and polymerization solvents for further synthesis of aniline-MIPs for selective extraction of aniline from contaminated water. GAUSSIAN 03 software was used to calculate the binding energy complexes between monomer-template. AAm as functional monomer and DVB as crosslinker using carbon tetrachloride (CCl_4_) as solvent was the system selected by theoretical calculations, and was then prepared by emulsion polymerization method using Span 80 as emulsifier and AIBN as initiator at 70 °C for 20 h. The copolymer produced showed high efficiency in aniline removal of CCl_4_.

GROMACS-molecular dynamics approach and computational screening to design rhodamine B (RhB)-MIP for selective removal from contaminated water was proposed in 2009 by Liu et al. [159]. Li et al. [160] reported a simple and cheap way to design of MIP–artificial antibodies with selective recognition of sulfadimidine (SM_2_) (a veterinary drug) using the same computational tools.

Dong et al. [161] also reported the development of a computational way to design acetochlor-MIP based on MD and quantum mechanics calculations (QM). Ahmadi et al. [162] reported a GAUSSIAN-computational approach for 3,4-methylenedioxymethamphetamine (MDMA)-MIP development.

Nicholls et al. [163] published a survey about theoretical and computational approaches for MIP design, where quantum chemical calculations, molecular dynamics, and statistic treatments of MIPs were explored. These topics are in line with the rapid growth of computer-based models applied to the evolution of MIP science, boosting the development of robust tools able to deal with many variables involved in the synthesis of a MIP, as well as to predict the best performance of the imprinted systems. Nicholls et al. [164] issued a critical review on theoretical and computational tools for rational design of biomimetic MIPs, where it is described the increasing use of computer-based models and software packages over the past decade, and the possibilities that derive from this, which includes the simulation of aspects of the complex molecular imprinting process.

Computational tools are undoubtedly a very active area and strong tool in the rational design of MIPs in line with the principles of the sustainable chemistry. They avoid time-consuming screening and experimental work, and therefore reduce cost and organic solvent utilization in MIP optimization.

## 3. Conclusions

Molecularly imprinted polymers can be obtained through a wide range of synthetic methods by conventional and emerging technologies. The increase in environmental awareness, and stricter regulation for the use of chemicals and economic competitiveness are challenging the scientific community and industry to explore greener strategies in their processes, preventing pollution, and reducing waste while maximizing the efficiency of the processes, and that can only be achieved by the application of the green chemistry and engineering principles. Molecular imprinting has much to gain in the application of these green tools, since new alternative solvents and clean technologies, combined with computational tools, can optimize both the polymer and the process itself, and enable an easier scale up, which could boost the use of MIPs at industrial scale.

## Figures and Tables

**Table 1:** Molecularly imprinted polymer (MIP) synthesis in supercritical carbon dioxide (scCO_2_).

Template	Strategy	Monomer	Crosslinker	Particle Size Diameter	Year	Ref.
Salicylic acid and acetylsalicylic acid	Non-covalent	DEGDMA	-	1.7 μm	2006	[22]
Propranolol	Non-covalent	MAA	DVB	100 nm	2006	[23]
Boc-l-tryptophan	Non-covalent	NIPAAm	EGDMA	micron-sized	2010	[24]
Flufenamic acid	Non-covalent	MAA NIPAAm	EGDMA	micron-sized	2011	[25]
Ibuprofen	Non-covalent	DMAEMA	EGDMA	~1 μm	2011	[26]
Bisphenol A	Non-covalent	MAA, FMMA	EGDMA	micron-sized	2012, 2018	[18,46]
Bisphenol A	Semi-covalent	BPADM	EGDMA	micron-sized	2012	[18]
Aspirin and Acetaminophen	Non-covalent	MAA 4-Vpy MMA	EGDMA	250–300 nm	2013	[30]
Bisphenol A and 2,4-dichlorophenoxyacetic acid	Non-covalent	MAA	EGDMA	300 nm	2013	[34]
Carbamazepine	Non-covalent	MAA	EGDMA	200 nm	2013	[35]
Labdanolic acid	Non-covalent	DMAEMA	EGDMA	micron-sized	2014	[38]
DBTSO_2_	Non-covalent	MAA	EGDMA	micron-sized	2014	[40]
Gallic acid	Non-covalent	MAA MMA	EGDMA	150–200 nm	2016	[41]
Acetamide	Non-covalent	MAA and MAM, ITA and HEMA	EGDMA	3.4–5.3 μm	2017	[43,47]

## Abbreviations

AAm	Acrylamide
AAP	Acetaminophen
ACET	Acetamide
ACN	Acetonitrile
AIBN	2,2′-Azobis(2-methylpropionitrile)
AME	Ametryn
API	Active Pharmaceutical Ingredient
AS	Aspirin
ATR	Atrazine
AuNPs	Gold nanoparticles
BAI	Baicalein
BENZ	Benzamide
BHb	Bovine hemoglobin
[Bmim][BF4]	1-butyl-3-methylimidazolium tetrafluoroborate
[Bmim][PF6]	1-butyl-3-methylimidazolium hexafluorophosphate
BPA	Bisphenol A
BPADM	Bisphenol dimethacrylate
BPO	Benzoyl peroxide
BSA	Bovine serum albumin
[Bvim]Br	1-Butyl-3-vinylimidazolium bromide
BZE	Benzoylecgonine
CAP	Chloramphenicol
CCl_4_	Carbon tetrachloride
CE	Cocaethylene
CFME	Carbon fiber microelectrode
CHO	Cholesterol
CMZ	Carbamazepine
COC	Cocaine
CPFX	Ciprofloxacin
CV	Cyclic voltammetry
2,4 D	2,4-Dichlorophenoxyacetic acid
DBTSO_2_	Dibenzothiophene sulfone
DEGDMA	Diethylene glycol dimethacrylate
DES	Deep Eutetic Solvents
DMAEMA	2-(Dimethylamino)ethylmethacrylate
DMF	Dimethylformamide
DMPA	2,2′-Dimethoxy-2-phenylacetophenone
DMSO	Dimethylsulfoxide
DNOC	4,6-Dinitro- *o*-cresol
DPA	Bisphenol acid
EA	Albumin egg
EGDMA	Ethylene glycol dimethacrylate
EIS	Electrochemical impedance spectroscopy
EME	Ecgonine methyl ester
ENRH	Enrofloxacin hydrochloride
E_2_	Estradiol
E_3_	Estriol
FA	Flufenamic acid
FF	Florfenicol
FMMA	Ferrocenylmethyl methacrylate
FO	Folic acid
GA	Gallic acid
GTFX	Gatifloxacin
HEMA	2-Hydroxyethyl methacrylate
HEX	Hexestrol
[Hmim][PF6]	1-Hexyl-3-methylimidazolium hexafluorophosphate
HPLC	High Performance Liquid Chromatography
HAS	Human Albumin Serum
HVA	Homovanillic Acid
*i*-NAM	Isonicotinamide
IAA	Indole-3-acetic acid
IBU	Ibuprofen
IF	Imprinting factor
ITA	Itaconic acid
ITO	Indium tin oxide
IVMA	Isovanillic acid
LA	Labdanolic acid
Lyz	Lysozyme
LOFX	Levofloxacin
MAA	Methacrylic acid
MBAAM	*N,N*-Methylene bisacrylamide
2-MBI	2-Macaptobenzimidazole
MDMA	3,4-Methylenedioxymethamphetamine
MHPE	4-Hydroxy-3-methoxyphenylethanol
MMA	Methyl methacrylate
MAM	Methacrylamide
1-MA-3MI-Br	1-(α-Methyl acrylate)-3-methylimidazolium bromide
MET	Metribuzin
MPS	3-(Trimethoxysilyl) propyl methacrylate
*n*-Bu_4_OH	Tetrabutylammonium hydroxide
NAM	Nicotinamide
4-NAP	2-Amino-4-nitrophenol
NIPAAm	*N*-Isopropylacrylamide
NMP	*N*-Methyl-2-pyrrolidone
NOFX	Norfloxacin
2-NP	2-Nitrophenol
4-NP	4-Nitrophenol
OA	Oleanolic acid
[Omim][PF6]	1-Methyl-3-octylimidazolium hexafluorophosphate
*o*-PD	*o*-Phenylenediamine dihydrochloride
OTA	Ochratoxin A
PA6	Polyamide-6
PAM	Picolinamide
*p*-BA	*p*-Hydroxybenzoic acid
pc	Critical pressure
PEDOT	Poly(3,4-ethylenedioxythiophene)
PGE	Pencil graphite electrode
*p*-HBA	*p*-Hydroxylphenylacetic acid
PHDFDMA	Poly(heptadecafluorodecyl methacrylate)
*p*-HPPA	*p*-Hydroxyphenylpropionic acid
PIV	Pivalamide
*p*-NA	Nitrophenyl acetate
Poly(DEGDMA)	Poly(diethylene glycol dimethacrylate)
PPy	Polypyrrole
PSMA	Poly(styrene- *co*-maleic acid)
PZFX	Pazufloxacin
QDs	Quantum Dots
Qu	Quercetin
RES	Resveratrol
RhB	Rhodamine B
scCO_2_	Supercritical carbon dioxide
SEM	Scanning electron microscopy
SPE	Solid phase extraction
SPME	Solid phase microextraction
St	Styrene
TBAH	Tetrabutylammonium hexafluorophosphate
TBAP	Tetrabutylammonium perchlorate
Tc	Critical temperature
TC	Tetracycline
TER	Terbutylazine
TFMAA	2-(trifluoromethyl)acrylic acid
*T*_g_	Glass transition temperature
THF	Tetrahydrofuran
THO	Theophylline
*T*_m_	Melting temperature
TMPTA	Trimethylolpropane acrylate
TRI	(2-Amino-4-methoxy-6-methyl-1,3,5)-triazine
TRIM	Trimethylpropane Trimethacrylate
Tyr	Tyrosine
URA	Uracil
VMA	Vanillylmandelic acid
2-Vpy	2-Vinylpyridine
4-Vpy	4-Vinylpyridine
